# Determination of progesterone compounds in the crude methanol extract of benalu duku leaves

**DOI:** 10.14202/vetworld.2019.358-366

**Published:** 2019-03-04

**Authors:** Lazuardi Mochamad, Bambang Hermanto, E. P. Hestianah

**Affiliations:** 1 Laboratory Veterinary Pharmacy, Faculty of Veterinary Medicine, Universitas Airlangga, Mulyorejo Rd., “C” Campus Surabaya, Surabaya 60115, Indonesia; 2 Department of Pharmacology, Faculty of Medical, Universitas Airlangga, Mayjen. Prof. Dr. Moestopo 47 Rd., “A” Campus Surabaya, Surabaya 60132, Indonesia; 3 Laboratory Histology, Faculty of Veterinary Medicine, Universitas Airlangga, Mulyorejo Rd., “C” Campus Surabaya, Surabaya - 60115, Indonesia

**Keywords:** Androgenic phyto progesterone, ^H^nuclear magnetic resonance of crude methanol extract, Isolation technique of progesterone, molecular fractions, progesterone compounds in crude methanol extract, progesterone-like effect

## Abstract

**Background and Aim:**

*Dendrophthoe* *pentandra* L. Miq (benalu duku) is a parasitic herb that commonly grows on the host plant *Lansium domesticum*. Researchers have found that the plant contains anticancer compounds and may contain phytoandrogens, including progesterone-like compounds, in its crude methanol extract. The objective of the current study was to investigate the compound of phyto progesterone in benalu duku leaves after extracted by methanol and prepared using an analytical column of high-performance liquid chromatography (HPLC).

**Materials and Methods:**

About 400 g of benalu duku leaves were pulverized, and their compounds were isolated by the isocratic method using an RP-18 analytical column (5 µm) with a mobile phase of 70:30 (methanol: water) in a photodiode array detector adjusted to 254 nm. The phyto progesterone compound was identified at a retention time of approximately 6.01 min.

**Results:**

By LC-electrospray ionization mass spectrometry focusing on molecular fractions, the fingerprint area of the Fourier transform-infrared spectroscopy (FT-IR, cm^−1^) and ^H^nuclear magnetic resonance (NMR) spectra indicated that the phyto progesterone product isolated was identical to the certified reference material of pure progesterone, particularly the specific functional groups in the FT-IR spectrum at wavenumbers of 1317.43 cm^−1^ and 1386.86 cm^−1^ and in the proton ^H^NMR spectrum at carbon 21 of progesterone (p<0.05).

**Conclusion:**

Each 49.888 µg/mL of crude benalu duku leaf extract dissolved in the mobile phase contained 28.515±0.713 µg/mL phyto progesterone.

## Introduction

Medicinal plants from around the world have contributed to almost 70% of therapeutic benefits in humans and animals. Benalu duku, for example, is a medicinal plant used as an alternative to synthetic cancer drugs [1-3]. Benalu duku is commonly known as a parasite of its host, *Lansium domesticum* [4,5]. The plant is distributed throughout the tropical and subtropical regions of Indonesia, Malaysia, the Philippines, and Thailand [6,7].

Benalu duku leaves are traditionally used to treat breast cancer and various other diseases involving cell proliferation [8-10]. Its crude methanol extract is reported to contain 0.033 mg L-asparagine, 0.017 mg L-threonine, 0.017 mg L-serine, 0.042 mg L-glutamine, 0.019 mg glycine, 0.018 mg L-alanine, 0.013 mg L-cysteine, 0.017 mg L-valine, 0.023 mg L-methionine, 0.018 mg L-isoleucine, 0.028 mg L-leucine, 0.01 mg L-tyrosine, 0.021 mg L-phenylalanine, 0.015 mg L-lysine, 0.008 mg L-histidine, 0.019 mg L-arginine, 0.021 mg L-proline, 0.009 mg L-hydroxyproline, 0.0003 mg L-hydroxylysine, and 0.007 mg ammonia per 4.385 g of leaves [4,11]. Benalu duku leaf infusion is nontoxic, especially in experimental animal models. Other compounds in the methanol extract of benalu duku leaves as detected by infrared spectrophotometry and thin-layer chromatography include hydroxyls, carbonyls, double-link carboxyls, and amine groups, in addition to alkaloids, flavonoid polyphenols, terpenoids, and steroids. In early 2013, researchers stated that crude methanolic extract from benalu duku leaves was predicted to contain plant hormone compounds for fertilization. Later research explained that crude methanolic extract from benalu duku leaves orally administered to female mice increased their progesterone levels [4,8,9]. Crude extract from benalu duku leaves obtained using maceration method dissolved in methanol was shown to contain four progesterone hormone derivatives with progesterone-like effects. The known active compounds that produce progesterone-like effects are steroid group of pregnane derivatives as prenanes, pregnandiene, and pregnanetriene. The pregnandiene and pregnanetriene are not strongest to stimulate progeterone-like effect. However, the prenanes is good to stimulate androgenic effect as progesterone, medroxyprogesterone acetate, megestrol acetate, and dydrogesterone [4,12].

This research aimed to obtain the active compound, progesterone or pregn-4-ene-3,20-dione (C_21_H_30_O_2_), after isolation by a modified preparation technique using high-performance liquid chromatography (HPLC) to analyze the compounds. The benefit of this research was that it obtained true compounds from HPLC separation through adsorption and partition from controlled chromatograms of the analyte peaks.

## Materials and Methods

### Ethical approval

This research has been approved by Ethics Commission of Veterinary Medicine, Universitas Airlangga with no. 1 KE 132.07.2018, date 31 July 2018 by Dean of Faculty of Veterinary Medicine, Universitas Airlangga.

### Certified reference material (CRM) progesterone and collection of plant material

**Figure-1:**
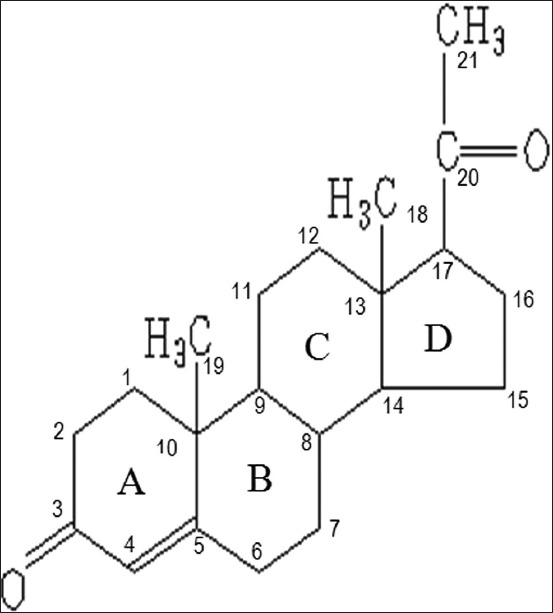
Molecular structure of progesterone or (8S,9S,10R,13S,14S,17S)-17-acetyl-10,13-dimethyl-1,2,6,7,8,9,10,11,12,13,14,15,16,17-tetradecahydro-3H-cyclopenta[a]phenanthren-3-one.

The progesterone standard for determining the bioactive progesterone compounds in the crude methanol extract of benalu duku leaves was obtained from Sigma-Aldrich Co., USA Lot No. SZBA321XV, with a molecular weight of 314.46, as shown in Figure-1. Benalu duku leaves were obtained from the Muara Enim district (south of Sumatra) and identified by the Research Center of Biology, Republic of Indonesia, at Raya Jakarta-Bogor Km. 46 Cibinong, West Java 16911, Indonesia. The benalu duku (*Dendrophthoe pentandra* L. Miq.) had been grown in *L. domesticum* for 5 years.

### Instrumental analysis

Instrumental analysis of the separated bioactive compounds from the matrix samples was conducted by HPLC using a Shimadzu CBM-20A Communication Bus Module to interact with a photodiode array detector in an ultraviolet-visible (UV-visible) M20A spectrometer, in which a LiChrospher^®^ 100 RP-18 column was fitted. The Fourier transform-infrared spectroscopy (FT-IR) instrument used was a Shimadzu IRAFFINITY-1S model at the scanning wavenumbers 500-4000 cm^−1^. The LC mass spectrometry (LC-MS) instrument was an Acella Triple Quadrupole Quantum Access Thermo with a Hypersil Gold 0.2 µM column of 10 cm in length. The nuclear magnetic resonance (NMR) instrument was a JEOL ECS-400, and analyses were performed using D_2_-grade methanol. All chemical reagents for HPLC, LC-MS, and sample preparation were of chromatographic and analytical grade.

### Preparation of methanolic extract of benalu duku leaves

The materials were cleaned, weighed to approximately 400 g, and pulverized; then, the powdered plant material was soaked in 1.0 L of absolute methanol for 24 h with occasional shaking. The mixture was filtered by a Buchner funnel and centrifuged at 3000 rpm for 15 min. The supernatants were collected in a specialized colored glass bottle and then dried under warm blowing nitrogen vapor (40°C). The soaking and filtration were repeated twice. The percentage yield of the dried extract was calculated. The extracts were stored in desiccators until further use [13,14].

### Research design

The research design was determined post-test only control groups, as described by Maraghehpour *et al*. [15] and Morrill *et al*. [16]. The total samples (n) were calculated as follows:


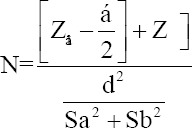


The Z_1_−α/2 at 5% significance was 1.96, and the Z_β_ at 5% error was 1.645. The tolerance of d was 3.62. The standard deviation of the sample trials (Sa) was 1.7, and the standard deviation of the control was 1.4. Equation 1 yielded an (N) of five research objects [4].

### Research protocol

The research protocol was performed as described below, following the steps listed in each section. Part 1: 400 g of pulverized benalu duku leaves were added to 1 L of methanol for the 1^st^ percolation and extraction by a moving method over 48 h. The supernatant was then collected in a specialized bottle. The extract was added to 1 L of methanol for the 2^nd^ percolation; then the 2^nd^ supernatant was collected. All supernatants were dried by a vacuum method, then collected, weighed, and stored in desiccators. The HPLC system was set to isocratic method parameters: Detection at 254 nm, a flow rate of 0.5 mL, stop time at 20 min, a mobile phase solution of 70% methanol:30% water, and a loop injection capacity of 40 µL. Part 2: The CRM for progesterone was dissolved (b/v) in the mobile phase solution at serial concentrations of 0.5, 0.75, and 1 µg/mL, then injected into the HPLC system in 0.5 µL aliquots. The benalu duku leaf crude extract was dissolved in the mobile phase solution, then filtered using a 0.20 µm filter and injected into the HPLC system. The chromatogram of the progesterone CRM versus those of the samples was compared using the retention time (RT) peak, and the progesterone concentrations in the samples were calculated. Part 3: Progesterone compounds in the matrix samples were isolated using the analyte column based on the CRM’s RT, and the waste product analyte was collected from the HPLC apparatus.

The isolated product was analyzed using infrared spectra to characterize the progesterone compounds after comparing them to the CRM, particularly at wavenumbers 500-1000 cm^−1^ and 1100-1750 cm^−1^. The isolated product was assessed mass spectrum fragmentation (molecular fractions [m/z]) of phyto progesterone and comparing to reference material of progesterone by LC-MS with electrospray ionization (ESI) MS [17]. The Acella parameters were set for autosampling at a capacity of 10 µL, column cleaning at 100 µL/s, and a stop time at 6 min determined by a vial sample capacity of 1.2 m. The column temperature was set at 22°C, and the pressure control was set at 10 bar. Part 4: The progesterone compounds isolated from the matrix samples were tested by proton NMR and compared with the proton NMR results for the progesterone CRM. Part 5: The data from sample testing versus the CRM was analyzed using MINITAB, version 17.0 at 5% significance, and the conclusion and recommendations were determined [18].

## Results

**Figure-2:**
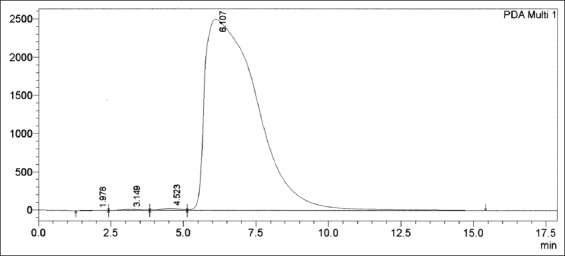
Progesterone certified reference material at 5 µg/mL in the mobile phase solution (methanol 70%:water 30%) at a wavelength of 254 nm, with a retention time of 6.107 min.

The crude extract from 400 g of pulverized benalu duku leaves yielded 40 g of matrix samples. The results indicated that RT of the progesterone CRM at 5 µg/mL by HPLC was 6.107 min (Figure-2).

**Figure-3:**
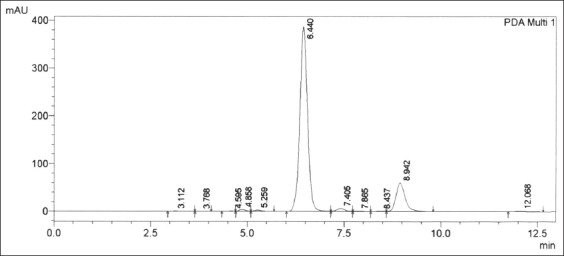
Chromatogram peaks from the crude methanol extract of benalu duku leaves dissolved in the mobile phase (methanol pro-high-performance liquid chromatography 70%:water pro-high-performance liquid chromatography 30%). The phyto progesterone was eluted at the retention time of 6.182 min.

Figure-3 shows that the benalu duku leaf crude extract dissolved in the mobile phase solution had three group peaks. The RTs were 4-6.5 min for the first group, 7.30-12.672 min for the second group, and 16.573-20.00 min for the third group.

**Figure-4:**
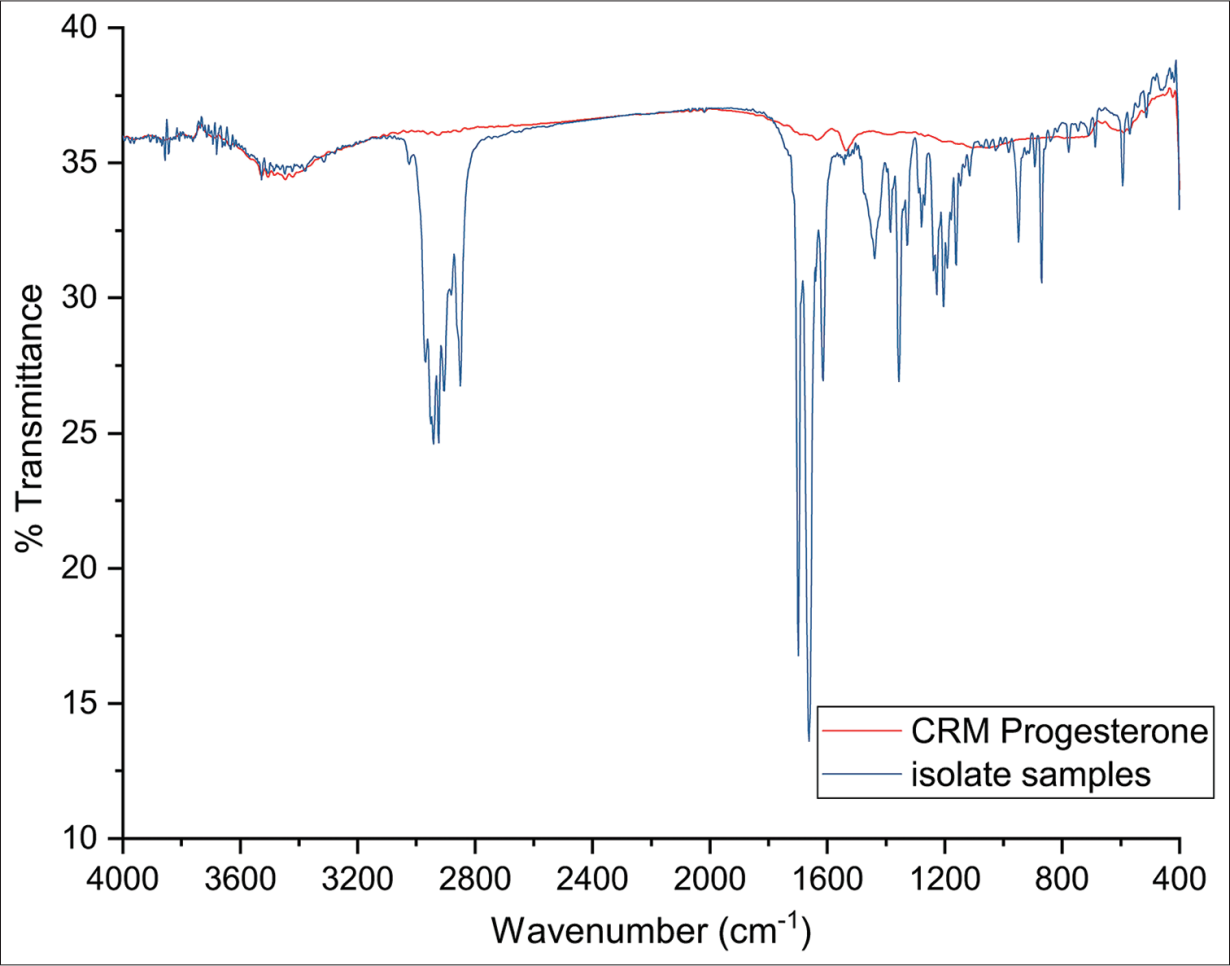
Infrared spectrum of the certified reference material (progesterone) by Fourier transform-infrared in KBr, with specific functional group peaks at wavenumbers 464.86 cm^−1^-949.01 cm^−1^ and 1161.19 cm^−1^-1699.34 cm^−1^.

Figure-4 shows the characterized infrared spectrum of the CRM in blue, while the isolated crude extract isolated from the benalu duku leaves is shown in red, with specific functional group peaks between wavenumbers 464.86 cm^−1^ and 949.01 cm^−1^ and 1161.19 cm^−1^ and 1699.34 cm^−1^. A non-specific peak presented between 2000 cm^−1^ and 4000 cm^−1^. The advantages of the infrared spectrum for CRM versus isolate from the benalu duku leaves are described in data obtained from Table-1.

**Table-1:** Interpretation of the infrared spectrum results for both the CRM and sample analytes.

CRM of progesterone			Isolate from crude methanol extract of benalu duku leaves		
	
Peak (cm^−1^)	Intensity	Compounds	Peak (cm^−1^)	Intensity	Compounds
594.100	34.150	H2C=O	590.24	36.115	H2C=O
1329.00	31.951	Single aromatic	1317.43	36.117	Single aromatic
1356.00	26.907	Single aromatic	1386.86	36.042	Single aromatic
1384.94	32.441	Ring of naphthalene (t)	None	None	None
1438.94	31.457	Ring of naphthalene (t)	1537.22	35.450	Ring of naphthalene (t)
1614.47	26.935	Ring of anthracene (s)	1633.76	35.849	Ring of anthracene (s)
1662.69	13.601	Ring of anthracene (s)			
1699.34	13.601	Ring of anthracene (s)	1689.70	36.045	Ring of anthracene (s)
None	None	None	2850.88	26.748	R-C-OH
None	None	None	2904.89	26.564	R-C-OH
2926.11	36.023	R-C-OH	2941.54	24.604	R-C-OH
None	None	None	2968.55	27.633	R-C-OH
3446.91	34.383	R-C-OH	3485.49	34.700	R-C-OH

t=twist, s=scissor, CRM=Certified reference material

**Figure-5:**
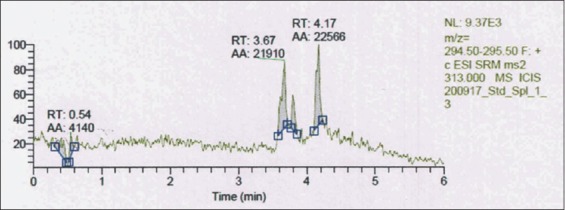
Liquid chromatography-electrospray ionization-mass spectrometry of the benalu duku leaf crude extract at 294.50-295.50 m/z. The ESI-MS2 313 retention time of 4.17 min and the chromatogram area of 22566 yielded a progesterone concentration of 28.268 µg/mL.

**Figure-6:**
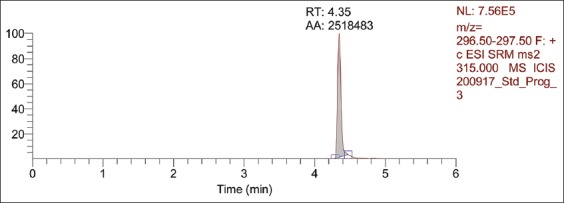
Liquid chromatography-electrospray ionization-mass spectrometry (ESI-MS) of the progesterone certified reference material at 50 µg/mL, with a chromatogram area of 39914 at the retention time of 4.18 min with 296.50-297.50 m/z and ESI-MS2 315.

Figure-5 shows that the isolated samples contained compounds similar to progesterone, as determined with reference to the CRM RT of 4.17 min with a 294.50-295.50 m/z ratios. Comparing the samples to the progesterone CRM in Figure-6 shows that at 296.50-297.50 m/z ratios, the ESI of CRM 315.000 was approximated to samples at ESI 313.000. The assessed area under the chromatogram of the benalu duku leaf crude extract of automatic area (AA) 22566 from Figure-5 was used to calculate the progesterone concentration based on the area under the chromatogram of the CRM, which was AA 39914 (Figure-6) at a 50 µg/mL concentration, yielding a 28.268 µg/mL concentration (Table-2, No. 1).

**Table-2:** Analysis of progesterone from five samples from the benalu duku leaf crude extract.

Crude methanolic extract of benalu duku leaves (µg/mL)	Concentration of progesterone (µg/mL)	CRM (µg/mL)
50.000	28.268^a^	29.010^b^
49.500	27.631^a^	30.100^b^
48.900	29.441^a^	28.090^b^
51.020	29.011^a^	29.310^b^
50.020	28.222^a^	29.380^b^

Superscripts a versus b in the same row indicate similarity at p>0.05 by the two-sample t-test.

**Figure-7:**
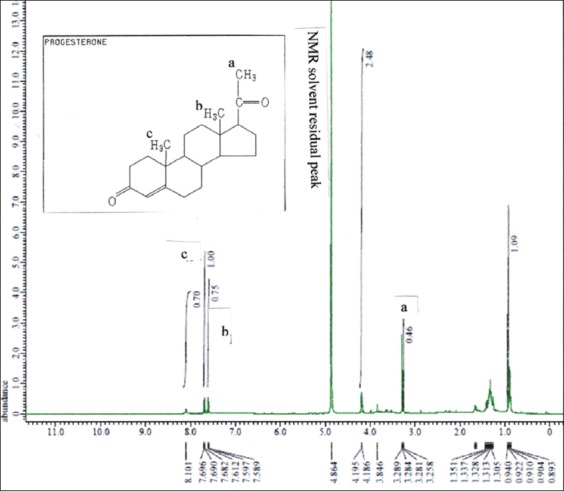
^1^H-nuclear magnetic resonance spectrum of the progesterone certified reference material dissolved in methanol and D_2_O at a field strength of 9.389766 T and 400 MHz, analyzed by a JEOL RESONANCE instrument. Letters a, b, and c indicate protons corresponding to carbon atoms 21, 18, and 19 of progesterone. The proton labeled “a” corresponds to an nuclear magnetic resonance solvent residual peak.

**Figure-8:**
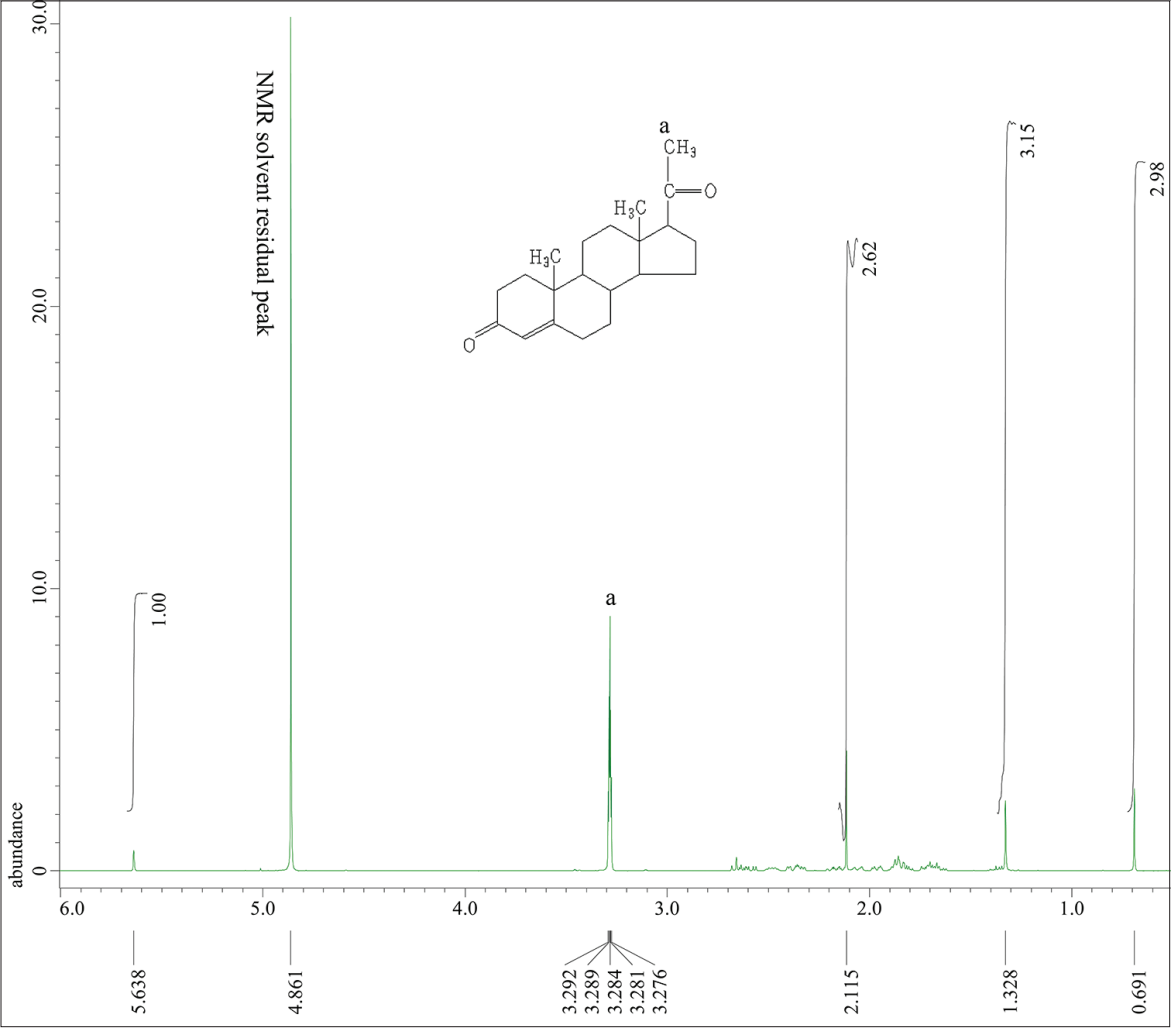
^1^H-nuclear magnetic resonance spectrum of the crude extract isolated from benalu duku leaves dissolved in methanol and D_2_O at a field strength of 9.389766 T and 400 MHz, analyzed by a JEOL RESONANCE instrument. The δ of 5.638 ppm was predicted to correspond to a proton in an aromatic ring.

The ^1^H-NMR results for the CRM dissolved in methanol and D_2_O are presented in Figure-7, and those of the benalu duku leaf crude extract are presented in Figure-8. Table-2 shows the phyto progesterone in the matrix isolate from the benalu duku leaf crude extract after separation by an analytical column.

## Discussion

The three peaks preceding those of the CRM (Figure-2) were predicted to be irrelevant and were identified as a noise peak, a turbulence effect from analyte entering the column, and another CRM component. The noise and turbulence peaks were easily identified. In practice, we can identify these peaks by triple or quadruple injection to test the stability of the peaks’ RTs and chromatogram areas. Peaks that do not stabilize are irrelevant. However, the peak from another CRM compound was highly stable and was often observed at the same RT in the same chromatogram area. Bioactive determination in biological matrices such as meat will essentially obtain an irrelevant peak as described above including the determination of β-agonist drugs in goats [18-23]. Figure-2 shows that progesterone was eluted at approximately 6.107 min, but the RT could easily drift by 2-4 min before and after 6.107 min. A drift of 2-3 min before 6.107 min would be found if the analyte had been dissolved in the mobile phase solution <30 min prior. A drift of 2-3 min would occur after 6.107 min if the analyte was dissolved in the mobile phase solution for >1 h. A decrease in pH would indicate that the RT of analyte had been >6.107 min after dissolved in the mobile phase for >1 h.

Phyto progesterone was eluted in the first group at the RT of 6.182 min (Figure-3). The RT of phyto progesterone apparently was more than RT of CRM at approximately drift of 0.075 min. Shifting RT indicates that the phyto progesterone molecule undergoes physicochemical change during dissolution in mobile phase. The phyto progesterone peak at the 6.182 min RT was then pooled from HPLC samples for identification by FT-IR, LC-ESI-MS, and proton NMR. The other phyto progesterone peaks were impurity peaks from other compounds in the crude matrix extract of the benalu duku leaves (4.220, 4.728, 5.091, 5.641, 6.837, 7.187, 9.317, 9.960, 14.133, 16.729, and 18.914 min). Other peaks from isolated compounds in the benalu duku leaf crude extract eluted at RTs from 7.300 to 12.672 min and 16.573 to 20.00 min were impurities peak and unrelated to compounds with progesterone-like effects, such as progesterone, medroxyprogesterone acetate, megestrol acetate, and dydrogesterone. The matrix compounds in this extract that eluted at RTs from 7.300 to 12.672 min and 16.573 to 20.00 min may be identified through more in-depth research.

Comparing the infrared CRM spectra with the benalu duku isolate (Table-1) was of interest because the overview shows that spectogram with the highest transmittance percent were found from 1300.00 cm^−1^ to 1700.00 cm^−1^. The predicted peak wavenumbers for the CRM at 1384.94 cm^−1^ and 1438.94 cm^−1^ were identical to those in the spectrum of the benalu duku isolate at 1317.43 cm^−1^ and 1386.86 cm^−1^, with high transmittance rates. The infrared spectrum indicated a single aromatic-ring with conjugated bonds. The infrared spectrum data described a single aromatic structure present at 1350.00 cm^−1^-1450.00 cm^−1^. Another peak on the infrared spectrum at 1689.70 cm^−1^-1699.34 cm^−1^ was identical between the CRM and the benalu duku isolate. These data indicated that wavenumbers 1680.00 cm^−1^-1700.00 cm^−1^ were scissor vibrations from a naphthalene ring (Figure-1, rings A and B or rings B and C). If this naphthalene ring was combined with the single aromatic ring (Figure-1, ring D), which was also indicated for the benalu duku isolate, the active substance was a molecule similar to phyto progesterone and the steroid, pregnane, derived at carbon bonds 1-21. Comparing the CRM’s infrared peak at 2926.11 cm^−1^ to the benalu duku extract isolate’s peak at 2941.54 cm^−1^ was unclear, although both peaks had high transmittance rates. However, the energies of the atoms in both peaks indicated involvement in the short bond structure of R-C-OH.

LC-ESI-MS is suitable for identifying unknown natural materials, especially by assessing their molecular weights. However, clarification is needed for testing using physicochemical-based instruments. The molecular mass of the active substance in the benalu duku extract isolate was identified as phyto progesterone (Table-2). The mean concentration of the benalu duku crude extract was 49.888 µg/mL, with a mean concentration of 28.515 µg/mL of phyto progesterone. This result is approximately 97.728% of the pure CRM concentration (mean of 29.178 µg/mL). Thus, ^H^NMR can be used to strengthen the LC-ESI-MS findings. Combining the two instruments produced mutually supportive interpretations [24-26]. Additional thermal analyses may be required to determine each substance’s melting point.

Figure-7 apparently that proton labeled “a” was identified to carbon atoms 21 of CRM at right of chemical shift NMR solvent residual peak (ppm). The carbon atoms 18 and 19 of CRM were left of chemical shift NMR solvent residual peak as labeled of “b” and “c” (ppm). Comparing to Figure-8 predicted that the proton NMR of the product isolated by the Lazuardi method might have contained protons from compounds other than progesterone [11,27-34]. The proton labeled “a” was a specific peak of carbon atom no 21 of progesterone molecule referred to Figure-1. However, at the right of chemical shift, NMR solvent residual peak in 2.115 ppm, 1.328 ppm, and 0.691 ppm was not specific protons from impurities compounds. Other impurities compound at δ of 5.638 ppm was predicted to correspond to a proton in an aromatic ring of impurities peak.

The LC-ESI-MS analysis indicated that part of the isolation process in running the HPLC from minutes 4 to 20 occurred as follows. From minutes 4 to 6.5, the eluted solution may have contained progesterone compounds; however, that isolate did not contain medroxyprogesterone acetate, megestrol acetate, or dydrogesterone. Table-2 shows that samples n=1-n=5 contained progesterone at approximately 28.515±0.713 µg/mL, as described below (p=0.189). The progesterone CRM described in Table-2 was apparently of a stable concentration, ranging from 28.08 µg/mL to 30.1 µg/mL [29-36].

## Conclusion

The phyto progesterone compound was found in the separation process between 4 and 6.5 min during the HPLC with an analytical column. The specific RT was detected at 6.107 min. Other compounds with progesterone-like effects were not found in the products separated from the benalu duku leaves. Future recommendations include exploring methanolic extract from benalu duku leaves to obtain other compounds with progesterone-like effects. The methanolic crude extract of benalu duku leaves with a mean concentration of 49.888 µg/mL after the adsorption-partition separation process contained 28.515±0.713 µg/mL of progesterone.

## Authors’ Contributions

LM collected plant material, performed the experiment, analyzed the data, and wrote the manuscript. BH and EPH participated in analyzing animal experiments after treatment. All authors read and approved the final manuscript.
